# Clinical Impact of Project ECHO in Children With Cancer in Western Kenya: A Case Series

**DOI:** 10.1200/GO-24-00279

**Published:** 2024-08

**Authors:** Gilbert Olbara, Festus Njuguna, Mary Ann Etling, Sandra Langat, Martha Kipng’etich, Charles N. Nessle, Gertjan J.L. Kaspers, Terry A. Vik, Tyler S. Severance

**Affiliations:** 1 Moi Teaching and Referral Hospital, Eldoret, Kenya; 2 Department of Child Health and Paediatrics, Moi University, Eldoret, Kenya; 3 Department of Pediatrics, Indiana University School of Medicine, Indianapolis, IN; 4 Richard M. Fairbanks School of Public Health, Indianapolis, IN; 5 Academic Model Providing Access to Healthcare, Eldoret, Kenya; 6 Department of Pediatrics, Division of Pediatric Hematology Oncology, University of Michigan, Ann Arbor, MI; 7 Fogarty International Center, National Institutes of Health, Bethesda, MD; 8 Emma Children’s Hospital, Amsterdam UMC, Vrije Universiteit Amsterdam, Amsterdam, the Netherlands; 9 Princess Máxima Center for Pediatric Oncology, Utrecht, the Netherlands; 10 Department of Pediatrics, University of Missouri, Columbia, MO

## Introduction

Pediatric oncology outcomes have improved significantly in high-income countries over the past 50 years with a survival rate of more than 80%.^1^ However, the majority of children with cancer worldwide are living in low-middle–income countries (LMICs), where the survival rate is typically <30%.^2,3^ This discrepancy is multifactorial, likely caused by delay in diagnosis, misdiagnosis, barriers to accessing care, treatment toxicity, and preventable relapse.^2–5^

Moi Teaching and Referral Hospital (MTRH), in partnership with Academic Model Providing Access to Healthcare (AMPATH) and Princess Máxima Center for Pediatric Oncology, is the only tertiary care hospital in western Kenya providing comprehensive cancer care for children.^6–8^ In 2019, only 250 pediatric malignancies were diagnosed in the region of 1,500 expected patients on the basis of the 20 million person catchment area, consistent with estimates from other LMICs.^9–12^

To address disparities in global childhood cancer outcomes, emerging literature has emphasized education as a mechanism for sustainable change.^13^ Project Extension for Community Healthcare Outcomes (ECHO) is a telehealth education platform delivered directly to local health care providers (HCPs) in resource-limited communities, currently with more than 6,700 programs in 205 countries and areas.^14,15^ Each ECHO program uses a hub-and-spoke model; the hub team consists of specialty experts and various local HCPs as spoke members. ECHO sessions meet regularly via videoconferencing, comprising a brief didactic lecture provided by a hub expert, a case presentation from a spoke member, and a facilitated discussion.^16^

In January 2020, the AMPATH Pediatric Oncology ECHO was launched at MTRH.^17^ These ECHO sessions allowed hub experts at MTRH to provide education and mentorship to spoke members located within the region to improve recognition, awareness, and diagnosis of malignancies in pediatric patients.^17^ Many ECHO programs focus on learner surveys and participation for evaluation; however, assessing the impact on patient outcomes is challenging.

Here, we highlight select patients shared in the ECHO program to demonstrate the evolving knowledge base of participants and the impact on patient care. These three patients were presented during the first 2 years of implementation, and informed consent was obtained from each participant’s guardian or legal representative. All aspects of the study were approved by the Moi Teaching & Referral Hospital/Moi University College of Health Sciences Institutional Research and Ethics Committee.

## Patient Discussion

### Patient 1

At the 15th ECHO session, a 3-year-old child with a chief complaint of abdominal swelling was presented by one of the spoke sites (Table 1). His symptoms progressed over several weeks, with associated watery stool, nonbloody, nonbilious vomiting, and fevers. On physical examination, he appeared ill with diffuse lymphadenopathy. His abdomen was grossly distended and tender with a palpable mass. His laboratory tests revealed anemia and mildly elevated blood urea and creatinine. The spoke member presenting the patient case considered further diagnostic evaluations but had uncertainty in the next step in diagnosis and management.

During the ECHO, the hub team experts raised the possibility of Burkitt lymphoma (BL). Given the presentation and differential diagnosis, further evaluations at the local center were deferred to facilitate urgent transfer to MTRH. Although rapid biopsy confirmed BL, he passed away during initial prephase chemotherapy because of complications of tumor lysis, a complication of BL treatment.

### Patient 2

At the 34th ECHO session, a spoke site presented a 5-year-old male with right-sided abdominal swelling associated with 4 months of weight loss, night sweats, and watery stools (Table 1). The physical examination included a large nontender abdominal mass. He was anemic, but other laboratory values were normal. The spoke site had concern for a malignancy and obtained an abdominal ultrasound and computed tomography scan, which were remarkable for a 10 × 8-cm mass located in the right pelvis with characteristics consistent with a lymphoma. At the local spoke site, a colonoscopy was performed with preliminary biopsy favoring BL.

The spoke members presented the patient case in the ECHO session to discuss the next best steps in management. The imaging and pathology reports from the outlying spoke site were reviewed, and the ECHO session participants—including the hematopathologist on the hub team—agreed with the diagnosis of BL. After the ECHO session, the hub team and spoke site coordinated the immediate transfer of the patient to MTRH for chemotherapy, which was completed without significant complications.

### Patient 3

At the 40th ECHO session, a spoke member presented a 12-year-old child with 4 months of progressive, worsening bilateral knee arthralgias (Table 1). He was admitted to the county hospital twice in the preceding 4 months for anemia requiring transfusions, but a cause had not been identified. On his third presentation, he was sick-appearing, pale, tachypneic, and edematous. His laboratory tests were notable for pancytopenia without peripheral blast cells.

In contrast to the previous patients, the spoke participant was concerned for malignancy and recognized the impending oncologic emergency. This prompted emergent referral to MTRH via communication with the hub team before the presentation at the ECHO session. The hub-and-spoke conversation led to a same-day transfer to MTRH where the patient was diagnosed with ALL. The patient case was still discussed at the ECHO session by the spoke member to highlight excellent medical decision making for educational purposes. After diagnosis, he tolerated induction chemotherapy well although he ultimately passed away while receiving consolidation chemotherapy because of infection.

## Discussion

For more than two decades, the ECHO model has bridged specialists with HCPs in medically isolated communities.^15^ The ECHO mission was to move knowledge, not patients to emphasize the educational benefit among HCPs, rather than its role in individual patient care.^18^ However, the impact on patient care is tangible, evidenced by the increasing number of new oncologic diagnoses at MTRH and the improved recognition of pediatric cancer in subsequent ECHO sessions.^12,17^ This case series demonstrates, in a narrative format, the improving medical decision making of spoke participants acquired by participation in the AMPATH Pediatric Oncology ECHO program.

The first patient presented with a rapidly enlarging, abdominal mass, but diagnostic uncertainty from the spoke site resulted in delayed evaluation, biopsy, and treatment. Because of this delay, the patient’s cancer continued to progress, and he died of tumor lysis syndrome and renal failure. Mortality during initiation of chemotherapy is significantly more common for patients with BL in LMICs, which emphasized the importance of early recognition to improve overall survival.^19–21^ In years past, this child would have likely died without a diagnosis.

Regarding the second patient, the spoke site providers were already suspicious of lymphoma, ordered diagnostic evaluations, and even obtained a biopsy at their hospital. The main question at the ECHO session was centered on referral, and management after the diagnosis was made. During the session, the hub team confirmed the diagnostic interpretation and the urgent need for chemotherapy. This prompt patient transfer and initiation of chemotherapy better positioned the patient to survive initial therapy.^19–21^

In the third scenario, the spoke site providers demonstrated early recognition of possible leukemia. They obtained diagnostic evaluations and facilitated referral through the hub team during ECHO case submission. The prompt recognition and management allowed for a more favorable induction course with a greater chance for long-term survival.^21^ This patient case also exemplifies another goal for ECHO programs to improve the confidence of providers in medically isolated communities as they often navigate early stages of disease independently.

**FIG 1.**
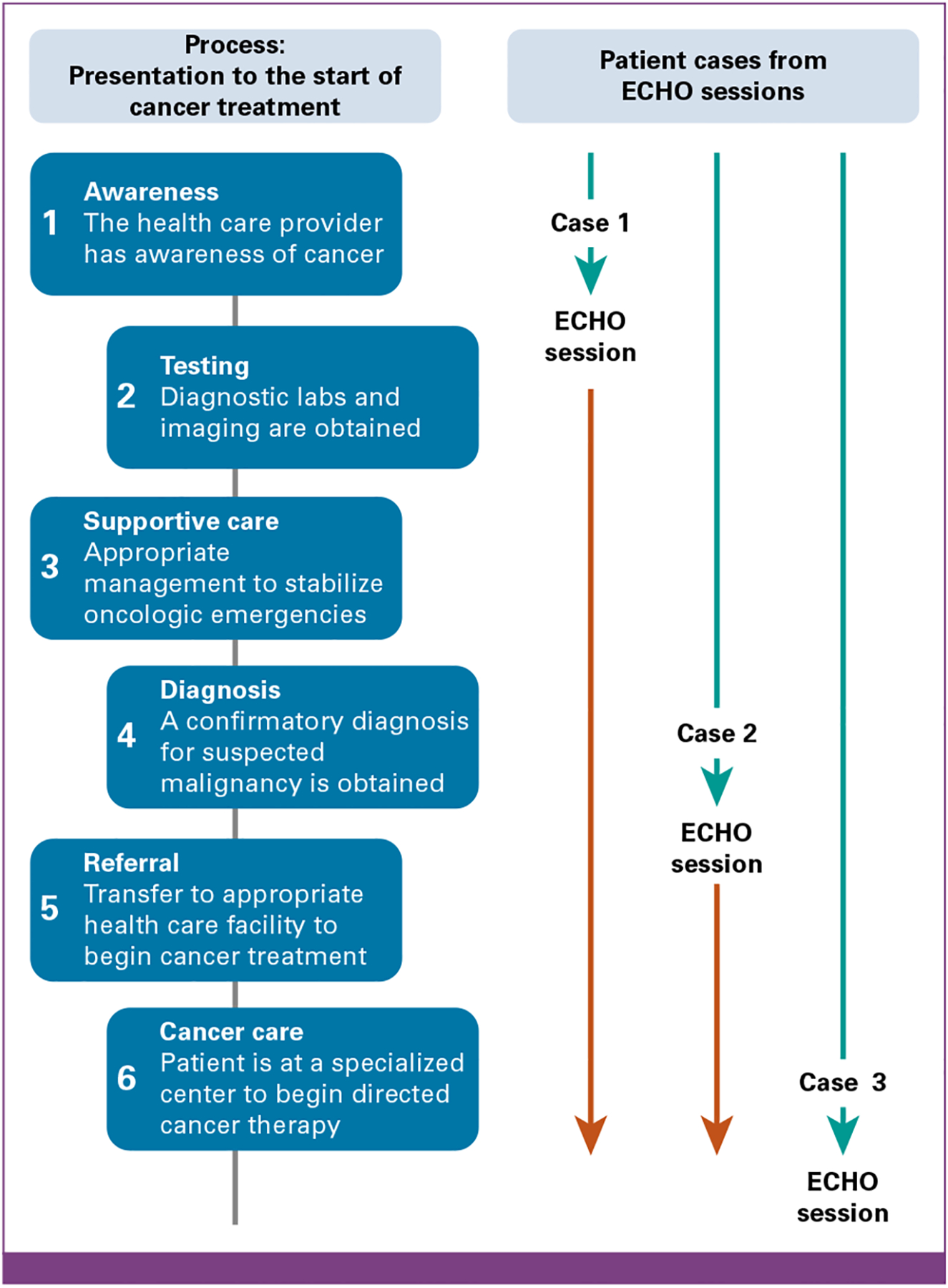
Visual representation of evolving clinical decision making among spoke participants regarding the diagnosis and early management of pediatric cancer. Teal arrows indicate management by spoke sites before ECHO session. Red arrows indicate management by cancer center after recommendations by the hub team after ECHO session. ECHO, Extension for Community Healthcare Outcomes.

The continuous educational impact was reflected not only in patient case discussions but also in the accelerating pace of diagnoses and treatment initiation. These patients narratively show the evolution of the medical decision making of spoke site participants: the first patient highlighted uncertainty in which diagnostic evaluations were performed, the second patient exhibited correct diagnostic evaluation but uncertainty in next steps in management, and the final patient demonstrated appropriate diagnostic tests which facilitated emergent patient transfer before the session (Fig 1). Through these patient presentations, specialists and HCPs were able to discuss and confirm next steps for evaluation, diagnosis, and treatment of their patients in real time.

This impact of ECHO on provider decision making is measurable.^18^ Although the primary objective of the AMPATH Pediatric Oncology ECHO is to increase the total number of patients diagnosed, prompt recognition allows for earlier treatment initiation, which can improve the outcomes in regions with limited access to health care.^5^ In the future, trends in early mortality and overall survival will be helpful metrics to follow in parallel with ECHO participation. Such data also demonstrate whether the goal of the WHO Global Initiative on Childhood Cancer is achieved.^22^

Given the increasing emphasis in the medical community on the potential impact of education in LMICs, the benefit highlighted here further supports the ECHO model as a costeffective and sustainable mechanism to improve care in resource-limited regions.^23–25^ Furthermore, Project ECHO is a powerful, telehealth education platform which may address major determinants in health disparities that contribute to delays in diagnosis and treatment.^12^ Clinicians should consider the continuous dissemination and replication of ECHO programs in LMICs to improve education and public health outcomes.

In conclusion, as the ECHO model continues to connect specialty experts with HCPs in medically isolated settings for education and partnership, patient care will continue to benefit. These three patients demonstrate how collaboration, learning, and mentorship within ECHO sessions can improve evaluation, diagnosis, and treatment of children with cancer in western Kenya.

## Figures and Tables

**TABLE 1.** Description of Clinical Characteristics of Individual Patient Cases Presented at Select ECHO Sessions

Case Component	Patient 1	Patient 2	Patient 3
ECHO session presented	15	34	40
Patient age, years	3	5	12
Chief complaint	Abdominal swelling	Abdominal swelling	Joint pain
Duration of symptoms	3 weeks	4 months	3–4 months
Associated symptoms	Watery stool, nonbloody, nonbilious vomiting, fevers	Weight loss, night sweats, watery stools	Fatigue, tachypnea, edema
Notable exam features	Distended, tender abdomen, LUQ mass, generalized lymphadenopathy	Large palpable nontender abdominal mass	Pallor, tachypnea, generalized edema
Laboratory tests	WBC 8.33 × 10^9^/L, ANC 5.0 × 10^9^/L, platelets 747 ×10^9^/L, Cr 63 μmol/L,^a^ urea 4.0 mmol/L	Hb 9.1 g/dL, WBC 8.64 × 10^9^/L, platelets 689 × 10^9^/L	Hb 2.8 g/dL, WBC 1.4 × 10^9^/L, ANC 0.7 × 10^9^/L, platelets 7 × 10^9^/L
Imaging	Abdominal CT	Abdominal ultrasound, abdominal CT	None
Final diagnosis	Abdominal BL^b^	Abdominal BL	Acute lymphoblastic leukemia
Outcome	Death; suspected tumor lysis syndrome during prephase	Survived; completed chemotherapy regimen	Death; invasive varicella infection on treatment

Abbreviations: ANC, absolute neutrophil count; BL, Burkitt lymphoma; Cr, creatinine; CT, computed tomography; ECHO, Extension for Community Healthcare Outcomes; Hb, hemoglobin; LUQ, left upper quadrant.

a Elevated on the basis of the laboratory reference range for age.

b Diagnosis not available until after ECHO discussion.
